# Effect of Lymphatic Invasion on Survival and Recurrence After Liver Transplantation in Patients with Hepatocellular Carcinoma and Its Prognostic Significance

**DOI:** 10.3390/diagnostics16050741

**Published:** 2026-03-02

**Authors:** Umut Tüysüz, İmam Bakır Batı, Tonguc Utku Yılmaz

**Affiliations:** 1 Department of General Surgery, Şişli Etfal Hamidiye Training and Research Hospital, Affiliated Hospital of Health Science University, Istanbul 34453, Turkey; 2 Department of Liver Transplantation, Bursa Acıbadem Hospital, Bursa 16210, Turkey; 3 Department of Organ Transplantation, Atakent Hospital, Acibadem Mehmet Ali Aydınlar University, Istanbul 34752, Turkey

**Keywords:** liver transplantation, lymphatic invasion, hepatocellular carcinoma, prognosis, recurrence

## Abstract

**Objective:** An important characteristic of Hepatocellular carcinoma (HCC) is that it features multicentric recurrences that can recur after curative treatment. Current recommended curative treatments for HCC include liver transplantation (LT), and prognostic evaluation and appropriate treatment selection are crucial in the management of HCC patients. Factors considered tend to include tumor size and number, lobar distribution, multinodularity, α-fetoprotein (AFP) level, degree of tumor differentiation, vascular invasion, and satellite nodules. However, the prognostic value of intrahepatic lymphatic vessel invasion (LVI) has rarely been reported for liver cancers. **Methods:** From January 2012 to December 2020, a total of 178 HCC patients who underwent liver transplantation consecutively were retrospectively enrolled. Those who underwent liver transplantation were divided into two groups based on the presence or absence of lymphatic vessel invasion. The primary aim was to compare the two groups for overall survival (OS), disease free survival (DFS), and recurrence rates, as well as to evaluate the prognostic effect of LVI after transplantation. **Results:** Poor tumor characteristics such as high tumor differentiation grade and MVI were significantly higher in the patient group with LI. Tumor recurrence and mortality rates were significantly higher in LI-positive recipients. **Conclusions:** The lymphatic invasion (LI) group displayed higher rates of recurrence and mortality. The findings emphasize the need to incorporate LI assessment into prognostic evaluations to enhance the management and outcomes of individuals with Hepatocellular carcinoma.

## 1. Introduction

Hepatocellular carcinoma (HCC) ranks fifth as the most common malignancy and is the third leading cause of cancer deaths worldwide [1]. Hepatocellular carcinoma (HCC) constitutes about 75–85% of all primary liver cancers. Although hepatectomy is the primary treatment method for HCC patients, liver transplantation (LT) is considered the optimal treatment for patients who have early HCC with decompensated cirrhosis and/or clinically significant portal hypertension [2]. An important characteristic of HCC is that it features multicentric recurrences that can recur after curative treatment. Prognostic evaluation and appropriate treatment selection are crucial in the management of HCC patients [3]. However, despite the strict and meticulous selection criteria applied to patients with HCC for liver transplantation, recurrence occurs in approximately 16% of recipients [4].

Over the last decade, several prognostic models have been suggested to estimate the risk of HCC recurrence after LT. These incorporate both biological and inflammatory features into selection criteria rather than simply relying on the tumor size and number pre- and post-LT [5]. Criteria that meet these models form the main axis guiding treatment decisions, thus improving patient survival and increasing the chance of LT success by reducing the risk of recurrence.

There are important clinic-pathological and radiological parameters for predicting patient survival and tumor recurrence following the LT for HCC. These often include tumor size and number, multinodularity, α-fetoprotein (AFP) level, degree of tumor differentiation, microvascular invasion (MVI), and satellite nodule [6]. However, the prognostic value of intrahepatic lymphatic invasion (LI) has rarely been reported for liver cancers.

Thus, our research aimed to assess the impact of LI on overall survival (OS), disease-free survival (DFS), and recurrence rates, as well as its prognostic influence post-transplantation.

## 2. Materials and Methods

This study included consecutive HCC patients who underwent deceased and living donor LT in the January 2012—December 2020 period. The patients were divided into the LI group (57 patients) and non-LI group (121 patients). The inclusion criteria were as follows: HCC patients (a) who underwent liver transplantation without major vascular invasion, (b) with pathological diagnosis of HCC after LT, and (c) with diagnosis of liver cirrhosis. Milan criteria were used for deceased donor liver transplantation (DDLT), while expanded criteria, along with downstaging modalities such as ablation and TACE, were used for living donor liver transplantation (LDLT) as acceptance criteria for HCC transplantation. In the LI group, 14 patients underwent DDLT and 43 patients underwent LDLT, while in the non-LI group, 48 patients underwent DDLT and 73 patients underwent LDLT.

Pathological characteristics were assessed based on the consensus of two experienced pathologists. LI was defined as the presence of tumor emboli or tumor cell in the lymphatic vessels in the surroundings of any tumor. Additional aspects were also assessed, including microvascular invasion (MVI), the histological grade of the tumor, and cirrhosis in the noncancerous liver tissue. All participants underwent postoperative follow-up, considering serum alpha-fetoprotein (AFP) levels, liver function test results, and imaging studies on the chest and abdomen. In the first two years, these assessments were performed every three months, followed by every six months thereafter. Standard immunosuppression was performed as previously described [7]. We identified patients who underwent successful downstaging or the bridging strategy as locoregional treatment. One patient received downstaging treatment and then underwent a living donor transplant after a one-month waiting period when there was no progression.

Post-transplantation recurrence of HCC was diagnosed based on imaging examinations revealing lesions suggestive of HCC recurrence after elevations in AFP were observed and biopsy confirmed the development of HCC after transplantation. DFS was defined as the time period from surgery to the first recurrence, metastasis, or last follow-up.

### Statistical Analysis

The mean, standard deviation, median, minimum, maximum, and percentage value frequency were used as descriptive statistics. The variable distributions were checked with the Kolmogorov–Smirnov test. An Independent Samples *t* test and Mann–Whitney U test were used to compare quantitative data, and the chi-square test to compare qualitative data. Kaplan–Meier (Log-Rank) was used for survival analysis, and SPSS 28.0 for statistical analyses.

## 3. Results

**Figure 1:**
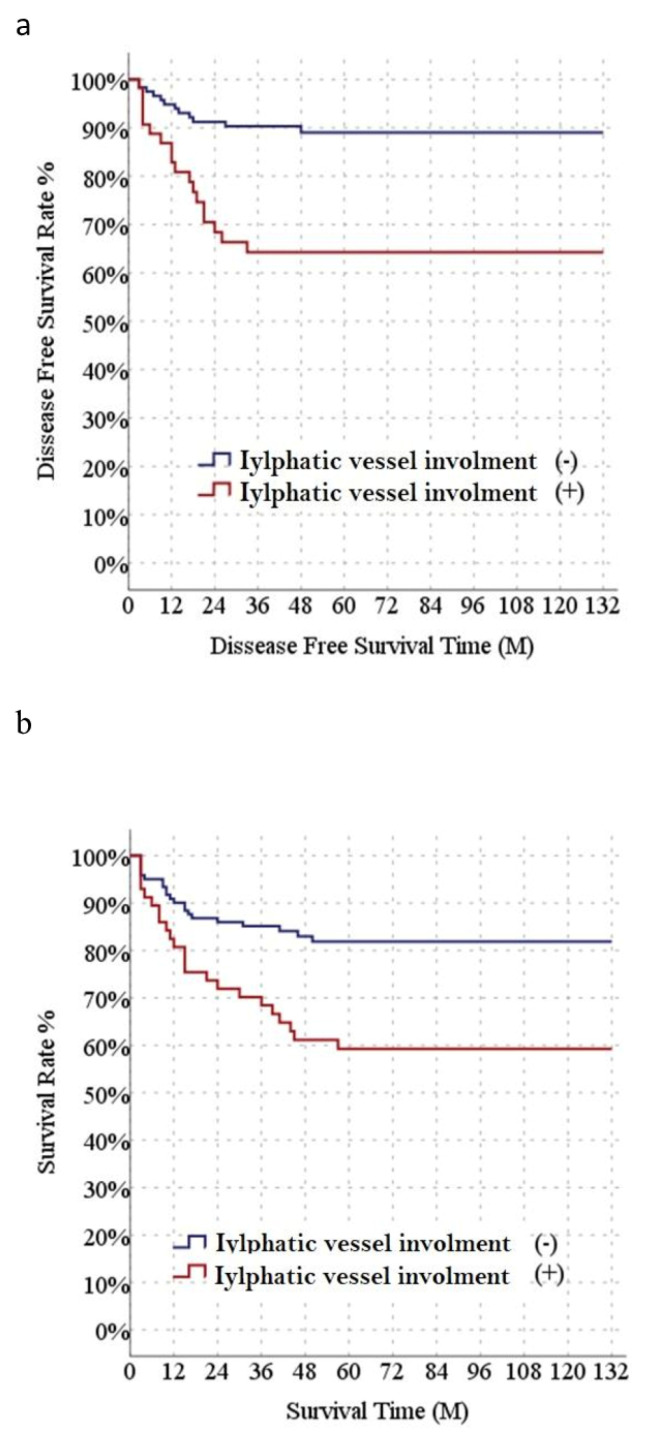
Comparison of long-term outcomes after liver transplantation between patients with or without lymphatic invasion. Kaplan–Meier curves of cumulative recurrence-free survival (**a**) and overall survival (**b**).

A total of 178 HCC patients who underwent LT were determined in the final analysis. Table 1 shows demographic and clinicopathological information and radiological findings of the included patients. The mean age, gender distribution, BMI, and MELD values of LI and non-LI groups were not significantly different. Conversely, the AFP value, tumor diameter (max), HCC lesion number, Child–Pugh B score were significantly higher in the LI group than non-LI group. The LI rate was higher in patients who received locoregional treatment. Also, poor tumor characteristics such as high tumor differentiation grade and MVI were significantly higher in the patient group with LI. Consistent with this, tumor recurrence and mortality rates were significantly higher in LI-positive recipients. There were no significant differences between the two groups for diabetes frequency or follow-up period (Table 1). Median disease-free survival (DFS) was significantly lower in the LI group than in the non-LI group (89.9 and 119.3 months, respectively) (Figure 1a). Median overall survival (OS) was significantly lower in the LI group (86.4 months) than the non-LI group (111.2 months) (Figure 1b). DFS values were significantly different between the non-LI (90.3, 84.9, 80.4, and 80.4%, respectively) and LI groups (80.6, 74.2, 63.8, and 63.8%, respectively) (Figure 1a). Survival rates between the non-LI (90.9, 85.1, 81.9, and 81.9%, respectively) and LI subgroups (82.5, 70.2, 59.2, and 59.2%, respectively) (Figure 1b).

**Figure 2:**
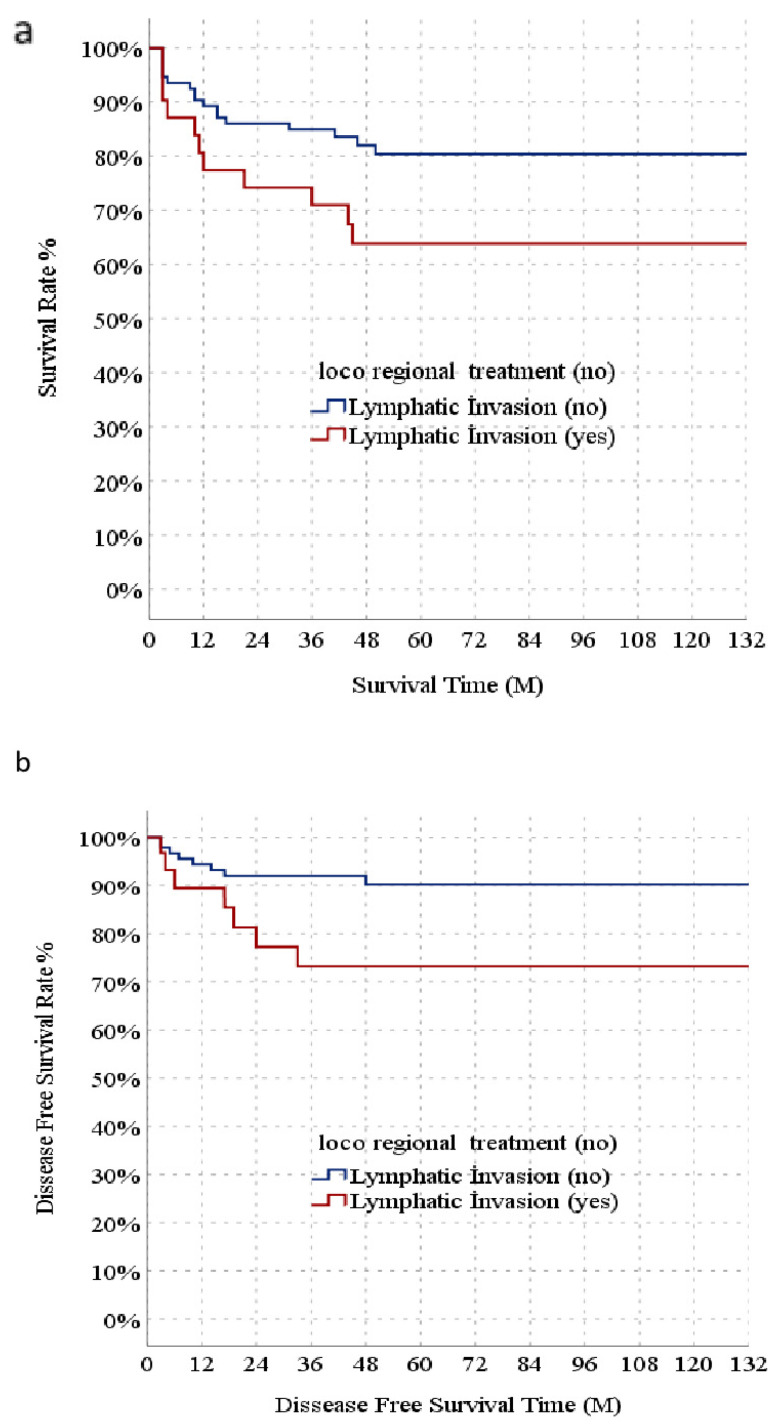
Comparison of long-term outcomes after liver transplantation between patients with or without lymphatic invasion in the subgroup who did not receive locoregional treatment. Kaplan–Meier curves of cumulative recurrence-free survival (**a**) and overall survival (**b**).

In the univariate model, age, max. tumor size (mm), number of HCC lesions, LRT, MVI, and LI had significant effects on DFS. Conversely, in the multivariate reduced model, max. tumor size (mm), number of HCC lesions, and LI had significant independent effects on DFS. In the univariate model, max. tumor size (mm), number of HCC lesions, LRT, MVI, and LI had significant effects on overall survival, whereas in the multivariate reduced model, max. tumor size (mm) and LI had significant independent effects on OS (Table 2). Recipients were stratified into a subgroup without LRT. The Child–Pugh B score and HCC lesion number were significantly higher in the LI group compared to in the non-LI group in the subgroup of patients who did not receive LRT. Tumor size (max) did not significantly differ in the two groups. In the subgroup of patients who did not receive locoregional therapy, intermediate stage tumor differentiation, MVI, recurrence, and mortality rates were significantly higher in the LI group than in the non-LI group (Table 3). In the patient subgroup who did not receive LRT, median DFS was significantly lower in the LI group than the non-LI group (100.9 and 120.6 months, respectively). Similarly, median OS was significantly lower in the LI group than in the non-LI group in the non-LRT subgroup. (90.8 and 109.7 months, respectively) (Figure 2b). With 1-, 3-, 5-, and 10-year DFS, there was a significant difference in the 1-, 3-, 5-, and 10-year rates between the non-LI group (94.3, 92.0, 90.1, and 90.1% respectively) and LI group (89.2, 73.0, 73.0, and 73.0%, respectively) in the non-LRT subgroup (Figure 2a). When comparing the OS of the LI and non-LI groups, there was a significant difference (94.8, 90.3, 89.0, and 89.0% vs. 86.7, 64.1, 64.1, and 64.1%, respectively) (Figure 2b).

## 4. Discussion

The lymphatic vessel (LV) system functions as the main pathway for cells providing antigens from the periphery to the lymph nodes, as well as maintaining tissue fluid homeostasis. Unlike other tissues, the liver has sinusoids instead of capillaries. Sinusoids are similar to lymphatic capillaries but different from blood capillaries. The lymphatic capillaries in the portal tract join into collecting vessels and then drain to the lymph nodes in the hepatoduedonal ligament [8]. Moreover, the lymphatic vessels around the central vein connect with 5–6 large lymphatic vessels. These extend towards the other side of the diaphragm, parallel to the inferior vena cava, towards the posterior mediastinal lymph nodes. The lymphatic fluid flowing under the capsule on the convex surface of the liver drains to the mediastinal lymph nodes via the coronary ligaments. Conversely, the lymphatic fluid flowing along the concave surface drains to the lymph nodes in the liver hilum and then to the regional lymph nodes [9,10,11]. It has been shown that cancer cells can enter lymphatic vessels and migrate to tumor-draining lymph nodes (TDLNs). Both cell autonomous and non-cell autonomous factors contribute to cancer cell entry into lymphatic vessels. The primary valve structures of the first lymphatic vessels (LCs) allow DCs to enter the cell in normal physiology and open when interstitial fluid pressure (IFP) exceeds lymphatic pressures in the vessel [12]. Tumors have high IFP, which facilitates the entry of cancer cells into lymphatic vessels at the tumor boundary. A very recent study showed that human malignant melanoma tumors with lymph node metastasis exhibit higher IFP and increased lymphatic vessel and microvascular density at the tumor periphery. Increased expression of vascular endothelial growth factor A (VEGF-A) and VEGF-C was also noteworthy here. As a result, peritumoral lymphangiogenesis is driven by the accumulation of VEGF-C, promoting lymph node metastasis [13]. VEGF-C and VEGF-D, released by tumor cells, bind to VEGFR-3 receptors on lymphatic endothelial cells (LECs). This interaction promotes the branching (sprouting) and dilation of lymphatic vessels. These factors not only increase the formation of new vessels but also improve the permeability of existing vessels, facilitating the intravasation of tumor cells. Proteolytically activated VEGF-C, in particular, can also accelerate this process through signaling via VEGFR-2. Chemokine CCL21, secreted by lymphatic endothelial cells, attracts tumor cells that carry the CCR7 receptor on their surface. This “chemotactic gradient” enables the directed migration of cells towards the lymphatic vessel. Tumor cells also enter the lymphatic lumen using specific “button-like” attachment points in the lymphatic vessels. Here, adhesion molecules such as LYVE-1 and VLA-4 stabilize the interaction of the cells with the endothelium. Along with these, immunosuppressive cytokines and cells (Treg, MDSC) transported to lymph nodes via lymphatic vessels suppress the immune response against the tumor. This local immunosuppression allows the tumor to recur [14]. In cirrhosis patients, portal pressure and sinusoidal blood flow increase with structural deformation around the portal and central vein. However, the number of lymphatic vessels increases [15]. A study showed that in liver fibrosis and cirrhosis, the delay in macromolecular blood changes in hepatocytes is associated with increased lymphatic drainage [16]. These observed changes in lymphatic vessels in cirrhotic patients with HCC might contribute to tumor progression and prognosis. However, further understanding of the relationship between cirrhosis, lymphatic vessels, and HCC can improve patient outcomes and guide therapeutic decisions. In addition to the impaired physiological mechanism in the lymphatic system, tumor cells may be prevented from reaching the lymph nodes due to structural deformation. This may explain the low lenfoid node metastasis (LNM) rate in patients with LI in HCC compared to intrahepatic cholangiocarcinomas (ICCs), which mostly develop in non-cirrhotic livers. One study showed an event specific to ICC: the presence of LI results in a negative prognostic effect on survival similar to that caused by LN involvement [17]. However, in our study, LN involvement was very low despite the LI rate, and only 1 out of 57 HCC patients with LI had LNM. This reflects the low LNM ratio, which is consistent with that observed in previous studies.

Many models have been proposed for predicting recurrence risk after LT for HCC [5]. They tend to have moderate predictive ability for clinical utility, and most use both pre- and post-LT clinical and histological parameters. Although considered advantageous in terms of accessibility, applicability, and convenience in clinical use, models using only pre-LT variables like the pre-MORAL and PLR scores have shown poor results in external validation [14]. Conversely, models that consider, for example, the R3 AFP score, UCLA nomograms, RETREAT score, and post-LT variables, have provided valuable prognostic information for predicting the risk of HCC recurrence. However, it is critical to note that these models are only capable of predicting average risk within a population rather than specific individual risk [18]. Therefore, individual tumor characteristics along with other clinical factors can significantly influence the risk of HCC recurrence after LT. In the HCC character structure, where there is heterogeneity even among multiple nodules, it is important to consider tools that improve personalized prediction ability rather than average population risk estimation.

However, no study has definitively demonstrated the superiority of one model over another in assessing the risk of recurrence. Using many tests without adjusting the *p*-value increases the likelihood of obtaining confusing results. It is necessary to consider recurrence risk factors not only pre- and post-LT use but also in their applicability and ease of use in clinical practice. It is also essential to consider factors such as data accessibility and the ability to different patient populations.

The most important disadvantage of LI, as well as of MVI and tumor differentiation, is that it cannot be detected before LT. Our study showed that determining lymphatic invasion provides an advantage in the early detection of hepatocellular carcinoma recurrence in patients who have undergone liver transplantation and enables the planning of effective strategies to prevent recurrence. However, in our study, the AFP value, tumor diameter (max), HCC lesion number, and MVI were significantly higher in the LI group. The presence of the abovementioned features before LT may suggest a relationship with LI.

Since the lymphatic system is related to immune system function, this may be dysfunctional due to cirrhosis. As a result, the tumor reduces the immune system’s ability to deal with cancer cells [15]. A recent study showed that mTOR inhibitors, Tacrolimus, and Mycophenolate mofetil did not affect HCC recurrence during the early period after LT [19,20]. However, we did not further evaluate the role of immunosuppression in HCC recurrence. The relationship between the LV system and metastasis is well known. However, although lymphatic capillary growth is observed in primary liver tumors, the role of this growth in the development and progression of these tumors is not well known. Lymphatic vessels play an important role in the pathogenesis of malignant tumors as one of the metastasis routes for tumor cells. The incidence of lymph node metastasis differs among tumors. This rate is 5.1% in HCC, and 45.1% in ICC. Conversely, another study reported that cancer cells resulted in LNM by entering older lymphatic vessels that already exist independently of lymphangiogenesis rather than entering the newly formed ones [21]. The portal LV is connected to the other portal tracts by short lateral branches. Therefore, lymphatic vessel invasion may have a clinical prognostic effect as strong as blood vessel invasion in liver cancer, especially in HCC. In terms of secondary liver cancers, few reports have evaluated the prognostic impact of intrahepatic LI in colorectal liver metastasis (CRLM) [22,23]. Similarly, LI has been reported as a major prognostic factor in malignancies such as breast, endometrial, and colon cancer [24,25]. A major concern for intrahepatic LI in HCC is the increased LNM incidence because there are numerous reports regarding the prognostic effect of LNM in HCC, especially in the hepatic hilum. The presence of LNM is considered a strong indicator for poor prognosis [26,27]. The relationship between LNM and LI and its mechanisms have also been inadequately examined [28]. Moreover, excluding the LI, peritumoral lymphangiogenesis was reported as a prognostic factor. In lymphangiogenesis, the lymphatic vessel count is determined via microscopy and used to determine lymphatic vessel density [29]. The incidence and prognostic effect of LI in HCC remains unclear. One study indicated recurrence-free survival in two patients (39 and 16 months) undergoing transplantation for HCC. The authors reported that LNM was not observed in the patients, concluding that LNI may not be a marker for poor prognosis [30]. Although lymph node metastasis is much lower in HCC with lymphovascular invasion than other tumors, it adversely affects total and disease-free survival and is a poor prognostic factor, reflecting the aggressive character of the tumor. For studies to continue investigating in this direction, it is essential to identify markers specific to LyECs that do not overlap with hepatocytes and LSECs. For example, high podoplanin expression, which is specific for LeYCs in tumor cells, has demonstrated the presence of a strong association with a high occurrence of poorly differentiated histopathological types. This suggests that podoplani plays a role in hepatocarcinogenesis. Moreover, it has been shown that both cirrhosis and LI are associated with high peritumoral lymphatic vessel density. The association of lymphangiogenesis with tumors has been indicated to play a role in tumor growth, aiding neovascularization and tumor invasion in HCC [31].

Lymphogenous and hematogenous spread are the two main forms of spread in cancer metastasis. The course of metastasis mostly depends on factors related to the peritumoral microenvironment, patient, and tumor. While hematogenous spread disseminates to distant areas after direct tumor cell entry into the blood vessels, tumor cells that penetrate the lymphatic vessels within the lymphogenous route are first transported to regional or distant nodes. They then enter the thoracic ductus, where they metastasize to distant areas through the subclavian vein [32]. Although HCC has advanced lymphangiogenesis, studies have been conducted to evaluate its relationship with intrahepatic LI, and naturally, lymph node metastasis remains insufficient. A study on CRLM patients showed the relationship between hepatic nodes and LI, revealing higher hepatic node involvement in LI-positive (23%) patients than in LI-negative (4%) ones. The authors suggested that in patients with LI, metastasis occurs in regional lymph nodes through the network of capsular, sublobular, and portal lymphatic vessels [33]. Several reports have shown that microscopic serosal invasion in HCC results in poor prognosis. Specifically, patients with serosal invasion tend to have a poor prognosis, due to not only the direct invasion of other organs but also microscopic serosal invasion [4,34]. However, the abovementioned studies did not evaluate the LI in HCC. Although it is very easy to consider LI as the main cause of LNM, the correlation between LI and LNM in HCC has not been indicated. This can be largely attributed to the low occurrence of lymph node metastasis (LNM) in HCC (0–7.45% in operable cases) [35,36]. Note that although research on tumor-related neovascularization drugs has specifically focused on angiogenesis for more than two decades, the absence of drugs that target it is an obstacle to the development of effective treatment due to our poor understanding of lymphangiogenesis in the liver. Understanding the specific mechanism and prognostic role of associated lymphangiogenesis and intrahepatic lymphatic invasion is crucial. Moreover, determining the target treatment for this is a critical necessity. More interestingly, in tumor-associated lymphangiogenesis, the prometastatic niche form is prepared by tumor cells in the early phase. Therefore, tumor-related lymphangiogenesis occurs in lymph nodes and distant organs before LNM. In fact, studies have demonstrated that lymphatics in distant organs, influenced by the progression of advanced cancer, experience structural changes [37,38]. The strength of our study results lies in that LI was confirmed using an adequate statistical method in a homogeneous manner. We analyzed a highly selected population treated under the same surgical policy and rigorous surgery quality control and determined that the possibility of LI increased in Child–Pugh B, MVI, and intermediate-state HCCs, in addition to tumors with high AFP levels and large tumor number and diameter. These are factors that adversely affect prognosis in HCC in general. At the same time, the incidence of LI (32%) was higher than that of MVI (23%) in non-advanced HCC patients who underwent transplantation. Moreover, LI patients had a high degree of MVI (73%). Significant differences were observed in both median survival and long-term OS and DFS in patients with LI. Considering that patients who did not receive LRT reflected the relatively early-stage patient group, excluding patients who received LRT from the analysis revealed that long-term OS and DFS were significantly reduced in the LI subgroup compared to the non-LI subgroup. Here, LI was found to be associated with parameters reflecting poor prognosis, such as a high AFP value, Child–Pugh B score, and number of HCC lesions, an intermediate stage, and MVI. It was observed that the high recurrence rate persisted in patients with LI. In both univariate and multivariate models, significant independent effects of LI were revealed on both OS and DFS. For the first time, our study showed that LI was associated with significantly reduced OS, DFS, and increased recurrence rates in patients undergoing transplantation for HCC. Moreover, we report for the first time that LI has an independent prognostic effect on HCC. Although robust scientific proof backing the cost-effectiveness of monitoring for HCC recurrence after transplantation is lacking, we believe that imaging evaluations linked to AFP should be conducted for transplant recipients every 3 to 6 months for a minimum of 5 years following the transplant. Under this context, examining the explant pathology can be beneficial in assessing the risk of HCC recurrence. From our perspective, the clinical significance of lymphovascular invasion may stem from the fact that HCC tends to recur primarily within the liver rather than metastasizing intra- and extrahepatic. In the context of adjuvant therapy, we focused on the evaluation of recurrence and also aimed to identify patient groups that could potentially benefit from adjuvant therapy. Patients with adverse findings, especially lymphatic invasion, appear to be strong candidates for adjuvant treatment. The potential role of adjuvant locoregional treatments such as TACE, radiofrequency ablation, and radiotherapy may be crucial for patients with high risk of intra- and extrahepatic metastasis and poor survival outcome. However, survival analysis based on adjuvant treatment was not conducted in this study, primarily due to potential selection bias and the limited number of patients who received adjuvant treatment.

The LV system in the liver remains an area wide open for examination. Further research on this topic could significantly advance our knowledge of liver physiology and patho-physiology. This study can provide a more comprehensive analysis of the implications of lymphatic vessel involvement in cirrhotic patients with HCC, shedding light on the clinical significance and potential avenues for further investigation in this complex interplay of factors. LI may be proposed as a potential predictor, its predictive value does not vary across different cohorts and populations. The results must be interpreted in a rigorous manner and evaluated as preliminary findings that can be for further validated in prospective randomized large studies. We propose future research avenues to further elucidate the mechanisms of lymphatic vessel involvement in HCC within cirrhotic liver environments. In particular, specific markers, signaling pathways, and therapeutic targets that could be crucial in improving patient outcomes should be investigated.

Limitations: This study’s retrospective design may have introduced bias and limited its ability to establish causal relationships between LI and outcomes, and it may have potentially overlooked certain confounding variables that were not accounted for during data collection. The relatively modest sample size of patients may have restricted the generalizability of the findings to a broader HCC population. Furthermore, due to the limited number of patients within the subgroups, analyses regarding LI might be especially underpowered, limiting the attainment of significant results. Larger sample sizes could provide more robust conclusions.

This study’s division of patients into the LI and non-LI groups based on retrospective data may have introduced selection bias, potentially influencing the observed outcomes.

As two experienced pathologists evaluated the pathological characteristics, inter-observer variability and subjectivity in assessing features like tumor differentiation and microvascular invasion could have impacted the accuracy and consistency of the results.

The follow-up duration, although outlined, may not have been sufficiently long to capture long-term outcomes accurately, such as late recurrences or metastases, which are crucial in assessing the impact of LI on survival. Novel biomarkers or imaging techniques that may provide additional prognostic information alongside LI assessment should be incorporated in future work, enhancing the predictive accuracy of the outcomes.

Future studies should aim to account for potential confounding variables more comprehensively, such as comorbidities, treatment variations, and lifestyle factors, to better isolate the impact of LI on HCC prognosis.

The present retrospective study was based on a relatively small population; however, it revealed comparable performance between the HCC patients with or without LI. Given that its impact on postoperative prognosis should not be underestimated, estimating the status of LI in patients with HCC before transplantation would help us determine those who would most benefit from liver transplantation. Accurately predicting LI using a noninvasive approach for patients with HCC is difficult prior to liver transplantation or surgical resection. LI can only be identified through microscopic analysis of surgical samples, which constrains its use, especially concerning preoperative treatment decisions. Therefore, the exploration of noninvasive methods that can determine LI status preoperatively is of great value. Future research can further elucidate the prognostic implications of LI in HCC patients undergoing liver transplantation, contributing to more informed clinical decision-making and improved patient outcomes.

## 5. Conclusions

This study’s findings reveal that patients with LI exhibited significantly reduced predicted survival periods and DFS compared to those without LI. The results underscore the prognostic relevance of LI across various stages of HCC, excluding advanced stages. LI was identified as a crucial independent prognostic indicator for recurrence, OS, and DFS in recipients of liver transplants for Hepatocellular carcinoma. This study highlights the importance of considering LI alongside established prognostic factors when evaluating and selecting appropriate treatments for HCC patients undergoing liver transplantation.

A recent study implied that prognostic importance of LI could potentially elucidate which subgroup of HCC can achieve maximal clinical benefit from specific novel therapies. Now, our study showed that LI is an independent prognostic factor in transplant recipients for HCC.

## Figures and Tables

**Table 1:** Demographic and clinical pathological characteristics of LI-positive and LI-negative patients.

		Lymphatic Invasion (No)				Lymphatic Invasion (Yes)				*p*	
		Mean ± sd/n%			Median	Mean ± sd/n%			Median		
Age (year)		58.3	±	8.2	60.0	57.2	±	9.7	59.0	0.601	^m^
Gender	Female	18		14.9%		11		19.3%		^X^ ^2^	
	Male	103		85.1%		46		80.7%			
BMI		26.4	±	3.7	26.0	26.8	±	3.7	27.0	0.465	^m^
Meld		12.2	±	6.1	10.0	12.3	±	5.6	11.0	0.689	^m^
AFP (ng/mL)		68.0	±	267.7	7.5	138.5	±	336.8	23.0	***0.001***	^m^
Tumor Diameter (mm)		31.1	±	20.0	25.0	41.1	±	23.4	40.0	***0.003***	^m^
Number Of HCC Lesions		2.2	±	2.2	1.0	3.6	±	3.4	3.0	***0.000***	^m^
Child–Pugh Score	A	60		49.6%		22		38.6%		***0.047***	^X^ ^2^
	B	57		47.1%		28		49.1%			
	C	4		3.3%		7		12.3%			
Locoregional Treatment	(no)	93		76.9%		31		54.4%		***0.002***	^X2^
	(yes)	28		23.1%		26		45.6%			
Tumor Differentiation Grade	well	20		16.5%		14		24.6%		***0.000***	^X2^
	moderate	53		43.8%		37		64.9%			
	advanced	48		39.7%		6		10.5%			
Microvascular Invasion	(no)	87		71.9%		16		28.1%		***0.000***	^X2^
	(yes)	34		28.1%		41		71.9%			
Diabetes Mellitus	(no)	92		76.0%		50		87.7%		0.070	^X2^
	(yes)	29		24.0%		7		12.3%			
Tumor Recurrence	(no)	109		90.1%		39		68.4%		***0.000***	^X2^
	(yes)	12		9.9%		18		31.6%			
Follow-Up Time (month)		59.9	±	31.6	64.0	60.1	±	38.8	71.0	0.735	^m^
Mortality	(no)	100		82.6%		34		59.6%		***0.001***	^X2^
	(yes)	21		17.4%		23		40.4%			

^m^ Mann–Whitney u test/^X2^ chi-square test.

**Table 2:** Uni- and multivariate analyses identify the risk factors for recurrence and mortality after liver transplantation for hepatocellular carcinoma.

Disease-Free Survival	Univariate Model					Multivariate Model				
	HR	% 95 GA			*p*	HR	% 95 GA			*p*
Age	0.95	0.92	-	0.99	***0.014***					
Gender	0.63	0.27	-	1.46	0.280					
BMI	0.99	0.89	-	1.10	0.832					
Meld	0.98	0.91	-	1.04	0.465					
AFP	1.00	1.00	-	1.00	0.714					
Max. Tumor Size (mm)	1.03	1.02	-	1.04	***0.000***	1.03	1.01	-	1.04	***0.000***
Number Of HCC Lesions	1.19	1.10	-	1.28	***0.000***	1.18	1.09	-	1.29	***0.000***
Child–Pugh Score	0.94	0.51	-	1.73	0.849					
Locoregional Treatment	2.32	1.13	-	4.75	***0.021***					
Tumor Differentiation grade	0.68	0.41	-	1.13	0.135					
Microvascular Invasion	10.40	3.63	-	29.83	***0.000***					
Diabetes Mellitus	0.83	0.32	-	2.18	0.709					
lymphatic İnvasion	3.76	1.81	-	7.81	***0.000***	2.39	1.11	-	5.13	***0.026***
**Overall Survival**	**Univariate Model**					**Multivariate Model**				
	**HR**	**% 95 GA**			***p***	**HR**	**% 95 GA**			***p***
Age	0.99	0.95	-	1.02	0.460					
Gender	1.05	0.47	-	2.35	0.908					
BMI	1.01	0.93	-	1.10	0.731					
Meld	1.00	0.96	-	1.06	0.855					
AFP	1.00	1.00	-	1.00	0.812					
Tumor Size (mm)	1.02	1.01	-	1.03	***0.003***	1.01	1.00		1.03	***0.021***
Number Of HCC Lesions	1.10	1.01	-	1.20	***0.022***					
Child–Pugh Score	1.41	0.88	-	2.26	0.154					
Locoregional Treatment	1.27	0.69	-	2.35	0.444					
Tumor Differentiation grade	0.76	0.50	-	1.16	0.202					
Microvascular Invasion	1.93	1.06	-	3.51	***0.031***					
Diabetes Mellitus	0.77	0.35	-	1.74	0.535					
Lymphatic Invasion	2.50	1.39	-	4.52	***0.002***	2.19	1.20		4.01	***0.011***
Cox Regression										

**Table 3:** Demographic and histopathological comparison between with and without lymphatic invasion in the subgroup without locoregional treatment.

		Lymphatic Invasion (No)				Lymphatic Invasion (Yes)				*p*	
		Mean ± sd/n%			Median	Mean ± sd/n%			Median		
Age		57.6	±	8.7	59.0	56.3	±	10.4	60.0	0.722	^m^
Gender	Female	13		14.0%		7		22.6%		0.259	^X^ ^2^
	Male	80		86.0%		24		77.4%			
BMI		26.2	±	3.8	26.0	26.9	±	3.6	26.0	0.338	^m^
Meld		12.8	±	6.3	12.0	14.4	±	6.2	13.0	0.131	^m^
AFP (ng/mL)		33.0	±	61.4	7.5	154.2	±	396.6	16.0	***0.009***	^m^
Tumor Size (mm)		31.0	±	20.4	25.0	37.5	±	24.5	32.0	0.192	^m^
Number Of HCC Lesions		2.0	±	1.7	1.0	3.5	±	2.8	3.0	***0.002***	^m^
Child–Pugh Score	A	41		44.1%		8		25.8%		***0.036***	^X2^
	B	48		51.6%		18		58.1%			
	C	4		4.3%		5		16.1%			
**Tumor Differentiation Grade**	**well**	**14**		**15.1%**		**8**		**25.8%**		***0.025***	^X2^
	**moderate**	**43**		**46.2%**		**19**		**61.3%**			
	**advanced**	**36**		**38.7%**		**4**		**12.9%**			
Microvascular Invasion	(no)	69		74.2%		11		35.5%		***0.000***	^X2^
	(yes)	24		25.8%		20		64.5%			
Diabetes Mellitus	(no)	73		78.5%		25		80.6%		0.799	^X2^
	(yes)	20		21.5%		6		19.4%			
Tumor recurrence	(no)	85		91.4%		24		77.4%		***0.039***	^X2^
	(yes)	8		8.6%		7		22.6%			
Follow-Up Time (month)		57.5	±	32.4	55.0	61.7	±	39.7	72.0	0.596	^t^
Mortality	(no)	76		81.7%		20		64.5%		***0.047***	^X2^
	(yes)	17		18.3%		11		35.5%			

^t^ Independent Samples *t* test/^X2^ chi-square test. ^m^ Mann–Whitney u test.

## Data Availability

All data generated or analyzed during this study are included in this article. Further enquiries can be directed to the corresponding author.
